# Supplementary material for: The Comparative Effects of Different Types of Oral Vitamin Supplements on Arterial Stiffness: A Network Meta-Analysis
Source: Nutrients. 2022 Feb 27;14(5):1009. doi: 10.3390/nu14051009 (PMC8912633; doi:10.3390/nu14051009)

Table S1. Search strategy for the MEDLINE database.

Adults<br>**OR**<br>Young adults<br>**OR**<br>Older adults<br>**OR**<br>Elderly adults<br>**OR**<br>Adult population<br>**OR**<br>Adults subjects	**AND**	Vitamins<br>**OR**<br>Oral vitamins supplementation<br>**OR**<br>Vitamin B9<br>**OR**<br>Folic acid<br>**OR**<br>Vitamin C<br>**OR**<br>Ascorbic acid<br>**OR**<br>Vitamin D<br>**OR**<br>Calciferol<br>**OR**<br>Vitamin D3<br>**OR**<br>Cholecalciferol<br>**OR**<br>Vitamin D2<br>**OR**<br>Ergocalciferol<br>**OR**<br>Vitamin E<br>**OR**<br>Tocopherol	**AND**	Arterial stiffness<br>**OR**<br>Aortic stiffness<br>**OR**<br>Pulse wave velocity<br>**OR**<br>PWv

Table S2. Quality grading of evidence.

Certainty assessment							№ of patients		Effect		Certainty	Importance
**№ of studies**	**Study design**	**Risk of bias**	**Inconsistency**	**Indirectness**	**Imprecision**	**Other considerations**	**Oral vitamin supplements**	**placebo/oral vitamin supplements**	**Relative (95% CI)**	**Absolute (95% CI)**		
Vitamin B9 versus Placebo												
**2**	randomised trials	serious[a]	not serious	not serious	not serious	none	25	25	-	SMD **0.14 SD lower** (0.69 lower to 0.42 higher)	⊕⊕⊕◯ Moderate	NOT IMPORTANT
Viamin C versus Placebo												
**2**	randomised trials	serious[a]	not serious	serious[b]	not serious	none	42	33	-	SMD **0.17 SD higher** (0.29 lower to 0.63 higher)	⊕⊕◯◯ Low	NOT IMPORTANT
Vitamin D versus Placebo												
**1**	randomised trials	serious[a]	not serious	not serious	not serious	none	39	40	-	SMD **0.04 SD lower** (0.56 lower to 0.47 higher)	⊕⊕⊕◯ Moderate	NOT IMPORTANT
Vitamin D2 versus Placebo												
**2**	randomised trials	serious[a]	not serious	not serious	not serious	none	132	132	-	SMD **0.24 SD lower** (0.5 lower to 0.01 higher)	⊕⊕⊕◯ Moderate	NOT IMPORTANT
Vitamin D3 versus Placebo												

**15**	randomised trials	serious[a]	not serious	not serious	not serious	none	1036	1037	-	SMD **0.08 SD lower** (0.24 lower to 0.08 higher)	⊕⊕⊕◯ Moderate	CRITICAL
Vitamin E versus Placebo												
**2**	randomised trials	serious[c]	not serious	serious	not serious	none	55	38	-	SMD **0.2 SD higher** (0.17 lower to 0.58 higher)	⊕⊕◯◯ Low	NOT IMPORTANT
Vitamin D3 versus vitamin D												
**1**	randomised trials	serious[a]	not serious	not serious	not serious	none	40	39	-	SMD **0.32 SD lower** (0.84 lower to 0.2 higher)	⊕⊕⊕◯ Moderate	NOT IMPORTANT
Vitamin D3 versus vitamin D2												
**2**	randomised trials	serious[a]	not serious	not serious	not serious	none	154	152	-	SMD **0.25 SD lower** (0.48 lower to 0.02 lower)	⊕⊕⊕◯ Moderate	NOT IMPORTANT

a. Mainly some concerns based on RoB2

b. The direction of effect in controversy

c. 50% high risk of bias based on RoB2

CI: confidence interval; **SMD:** standardised mean difference

Table S3. Effectiveness ranking of different types of oral vitamin supplements on arterial stiffness.

	Rank statistics			Probabilities	
	**Mean**	**Median**	**95%CIs**	**Best**	**SUCRA**
**Placebo**	4.0	5.0	1.0-7.0	0.01	0.50
**Vitamin B9**	3.3	4.0	1.0-5.0	0.31	0.61
**Vitamin C**	5.5	6.0	1.0-7.0	0.05	0.24
**Vitamin D**	3.9	4.0	2.0-4.0	0.14	0.52
**Vitamin D2**	3.4	3.0	2.0-5.0	0.20	0.60
**Vitamin D3**	2.3	4.0	1.0-7.0	0.26	0.78
**Vitamin E**	5.5	6.0	1.0-7.0	0.03	0.25

Table S4. Heterogeneity statistics for each comparison.

	**Q (df)**	**I^2**	**τ^2**	**p**
**Vitamin B9 vs Placebo**	0.09 (1)	0.00 %	0.00	0.76
**Vitamin C vs Placebo**	0.09 (1)	0.00%	0.00	0.76
**Vitamin D vs Placebo**	0.00 (0)	-	0.00	-
**Vitamin D2 vs Placebo**	0.28 (1)	0.00 %	0.00	0.60
**Vitamin D3 vs Placebo**	40.94 (15)	63.40%	0.06	0.00
**Vitamin E vs Placebo**	2.03 (3)	0.00%	0.00	0.57
**Vitamin D3 vs Vitamin D**	0.00 (0)	-	0.00	-
**Vitamin D3 vs Vitamin D2**	0.56 (1)	0.00%	0.00	0.46

Table S5. Subgroup analysis according to mean age (<65 years or >65 years) by type of vitamin on arterial stiffness.

	**Adults <65 years**				**Older Adults >65 years**			
	**n (samples)**	**ES (95%CIs)**	**I^2**	**%Change (m/s)**	**n (samples)**	**ES (95%CIs)**	**I^2**	**%Change (m/s)**
**Vitamin B9 vs Placebo**	2 (2)	-0.14 (-0.69, 0.42)	0.00%	-26.0%	-	-	-	-
**Vitamin C vs Placebo**	2 (2)	0.17 (-0.29, 0.63)	0.00%	48.0%	-	-	-	-
**Vitamin D vs Placebo**	-	-	-	-	1 (1)	-0.04 (-0.56, 0.47)	-	-
**Vitamin D2 vs Placebo**	2 (2)	-0.24 (-0.50, 0.01)	0.00%	-44.0%	-	-	-	-
**Vitamin D3 vs Placebo**	5 (5)	-0.26 (-0.61, 0.09)	66.10%	-71.0%	8 (11)	-0.01 (-0.15, 0.13)	40.80%	-15.0%
**Vitamin E vs Placebo**	1 (3)	-0.04 (-0.57, 0.50)	0.00%	-3.0%	1 (1)	0.43 (-0.10, 0.96)	-	-
**Vitamin D3 vs Vitamin D**	-	-	-	-	1 (1)	-0.32 (-0.84, 0.20)	-	-
**Vitamin D3 vs Vitamin D2**	1 (1)	**-0.30 (-0.57, -0.03)**	-	-	1 (1)	-0.10 (-0.55, 0.35)	-	-

Table S6. Subgroup analysis according to length of intervention by type of vitamin on arterial stiffness.

	Intervention <12 weeks				Intervention >12 weeks			
	**n (samples)**	**ES (95%CIs)**	**I^2**	**%Change (m/s)**	**n (samples)**	**ES (95%CIs)**	**I^2**	**%Change (m/s)**
**Vitamin B9 vs Placebo**	2 (2)	-0.14 (-0.69, 0.42)	0.00%	-25.0%	-	-	-	-
**Vitamin C vs Placebo**	2 (2)	0.17 (-0.29, 0.63)	0.00%	48.0%	-	-	-	-
**Vitamin D vs Placebo**	-	-	-	-	1 (1)	-0.04 (-0.55, 0.47)	-	-
**Vitamin D2 vs Placebo**	-	-	-	-	2 (2)	-0.24 (-0.50, 0.01)	0.00%	-45.0%
**Vitamin D3 vs Placebo**	3 (3)	0.34 (-0.30, 0.98)	80.60%	11.0%	11 (13)	**-0.15 (-0.30, -0.00)**	53.10%	-60.0%
**Vitamin E vs Placebo**	2 (4)	0.20 (-0.17, 0.58)	0.00%	-3.0%	-	-	-	-
**Vitamin D3 vs Vitamin D**	-	-	-	-	1 (1)	-0.32 (-0.84, 0.20)	-	-
**Vitamin D3 vs Vitamin D2**	-	-	-	-	2 (2)	**-0.25 (-0.48, -0.02)**	0.00%	-52.0%

Table S7. Subgroup analysis according to type of pulse wave velocity by type of vitamin.

	Central PWv				Peripheral PWv			
	**n (samples)**	**ES (95%CIs)**	**I^2**	**%Change (m/s)**	**n (samples)**	**ES (95%CIs)**	**I^2**	**%Change (m/s)**
**Vitamin B9 vs Placebo**	2 (2)	-0.14 (-0.69, 0.42)	0.00%	-25.0%	-	-	-	-
**Vitamin C vs Placebo**	-	-	-	-	2 (2)	0.17 (-0.29, 0.63)	0.00%	48.0%
**Vitamin D vs Placebo**	1 (1)	-0.04 (-0.56, 0.47)	-	-	-	-	-	-

**Vitamin D2 vs Placebo**	2 (2)	-0.24 (-0.50, 0.01)	0.00%	-43.0%	-	-	-	-
**Vitamin D3 vs Placebo**	13 (15)	-0.10 (-0.26, 0.07)	64.40%	-85.0%	1 (1)	0.25 (-0.31, 0.81)	-	-
**Vitamin E vs Placebo**	2 (4)	0.20 (-0.17, 0.58)	0.00%	55.0%	-	-	-	-
**Vitamin D3 vs Vitamin D**	1 (1)	-0.32 (-0.84, 0.20)	-	-	-	-	-	-
**Vitamin D3 vs Vitamin D2**	2 (2)	**-0.25 (-0.48, -0.02)**	0.00%	-36.0%	-	-	-	-

Table S8. Subgroup analysis according to type of vitamin (water-soluble or fat-soluble) on arterial stiffness.

**Water-soluble vitamins**				**Fat-soluble vitamins**			
**n (samples)**	**ES (95%CIs)**	**I^2**	**%Change (m/s)**	**n (samples)**	**ES (95%CIs)**	**I^2**	**%Change (m/s)**
4 (4)	0.04 (-0.31, 0.40)	0.00%	17.0%	17 (22)	-0.09 (-0.21, 0.02)	47.20%	-73.0%

Table S9. Meta-regression according to mean age and length of intervention for vitamin D3 vs Placebo on arterial stiffness.

Vitamin D3 vs Placebo	Coefficient	95%ICs	P value
Mean age	0.01	-0.01, 0.03	0.235
Length	-0.01	-0.03, 0.02	0.622

Figure S1. Quality assessment using the Cochrane Collaboration's tool for assessing risk of bias in randomized clinical trials (RoB2) for each study.

Reference	D1	D2	D3	D4	D5	Overall
Mangoni et al, 2002	+	!	+	+	+	!
Mangoni et al, 2005	+	!	+	+	+	!
Nightingale et al, 2003	!	!	!	!	+	!
Nightingale et al, 2007	!	!	+	!	+	!
Dreyer et al, 2014	+	+	!	!	+	!
Kovesdy et al, 2012	+	!	+	!	+	!
Forouhi et al, 2016	+	!	+	+	+	!
Larsen et al, 2012	+	+	!	+	+	!
Marckmann et al, 2012	+	+	+	+	+	+
Hewitt et al, 2013	+	+	+	!	+	!
Witham et al, 2013	+	+	+	+	+	+
Mose et al, 2014	+	+	!	+	+	!
Pilz et al, 2015	+	!	+	+	+	!
Witham et al, 2015	+	+	!	+	+	!
Bressendorff et al, 2016	+	+	!	!	+	!
Kumar et al, 2017	+	+	+	+	+	+
Sluyter et al, 2017	+	!	!	+	+	!
Gepner et al, 2012	+	!	+	+	+	!
Levin et al, 2017	+	!	!	!	+	!
Tomson et al, 2017	+	+	+	+	+	+
Rasool et al, 2006	!	!	+	!	+	!
Stonehouse et al, 2016	+	+	+	+	+	+

+ Low risk

! Some concerns

- High risk

D1 Randomisation process

D2 Deviations from the intended interventions

D3 Missing outcome data

D4 Measurement of the outcome

D5 Selection of the reported result

Figure S2. Overall quality assessment using the Cochrane Collaboration's tool for assessing risk of bias in randomized clinical trials (RoB2).

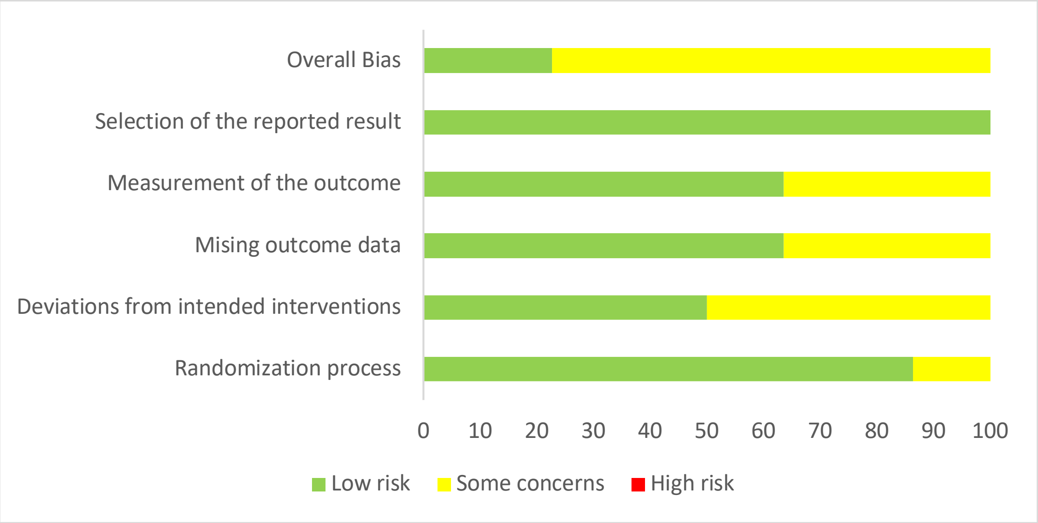

Figure S3. Rankogram for each of different types of oral vitamin supplements on arterial stiffness.

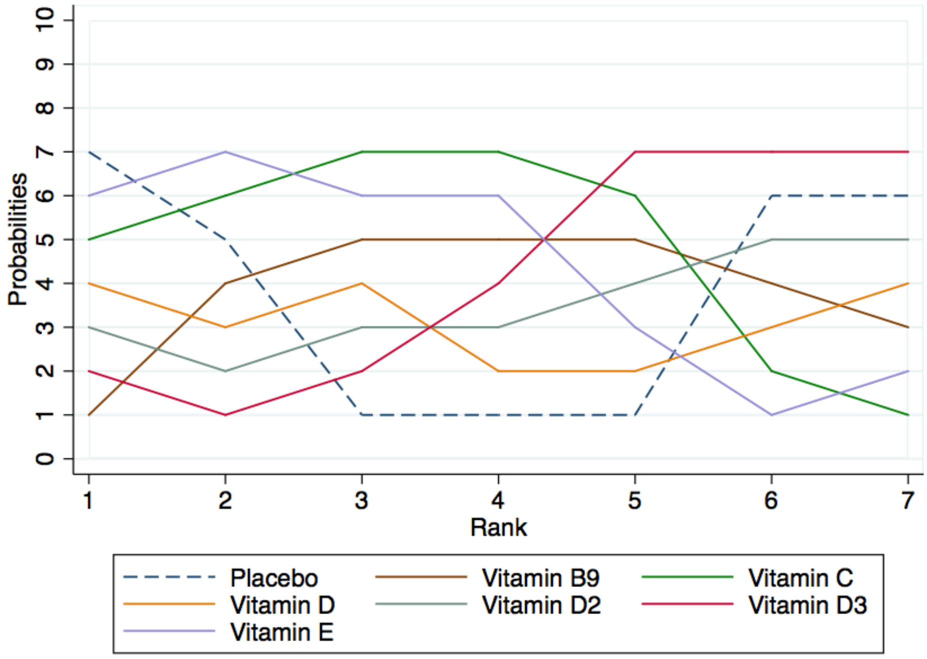

Figure S4. Funnel plot for comparison-specific pooled mean differences.

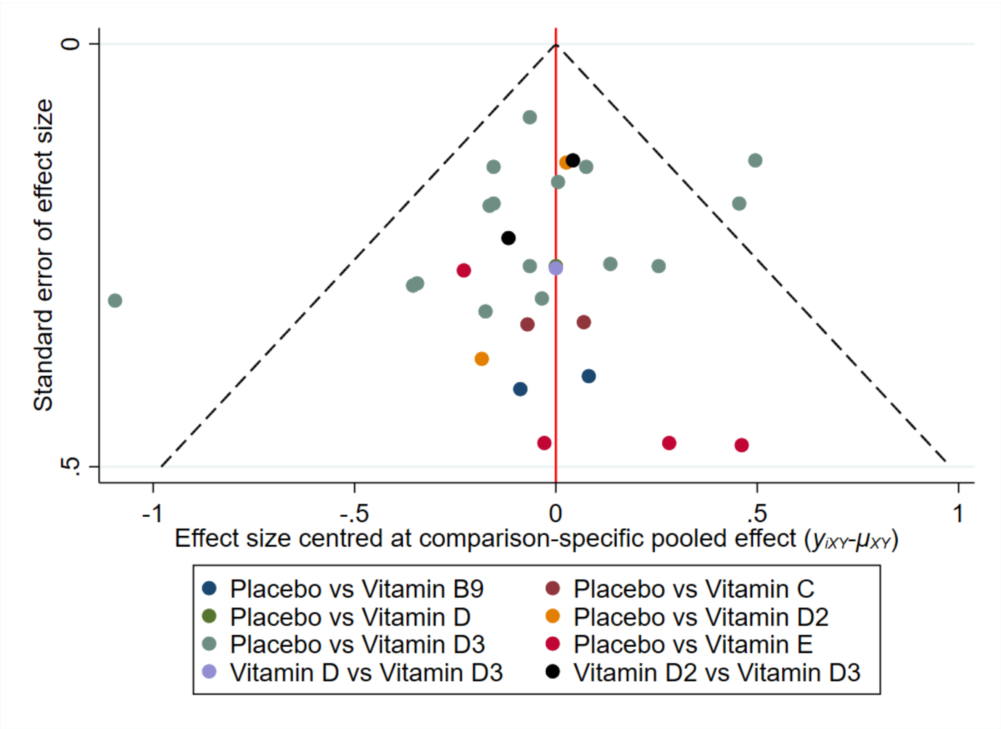

Supplement: Supplementary file 1 [file nutrients-14-01009-s001.zip › nutrients-1608500-supplementary.pdf]
